# Compressed Deep Learning Models for Wearable Atrial Fibrillation Detection through Attention

**DOI:** 10.3390/s24154787

**Published:** 2024-07-24

**Authors:** Marko Mäkynen, G. Andre Ng, Xin Li, Fernando S. Schlindwein, Timothy C. Pearce

**Affiliations:** 1 Biomedical Engineering Research Group, School of Engineering, University of Leicester, Leicester LE1 7RH, UK; mm915@leicester.ac.uk (M.M.); xin.li@leicester.ac.uk (X.L.); fss1@leicester.ac.uk (F.S.S.); 2 National Institute for Health Research Leicester Cardiovascular Biomedical Research Centre, Glenfield Hospital, Leicester LE5 4PW, UK; gan1@leicester.ac.uk; 3 Department of Cardiovascular Sciences, University of Leicester, Leicester LE1 7RH, UK

**Keywords:** atrial fibrillation, artificial intelligence, deep learning, wearable devices

## Abstract

Deep learning (DL) models have shown promise for the accurate detection of atrial fibrillation (AF) from electrocardiogram/photoplethysmography (ECG/PPG) data, yet deploying these on resource-constrained wearable devices remains challenging. This study proposes integrating a customized channel attention mechanism to compress DL neural networks for AF detection, allowing the model to focus only on the most salient time-series features. The results demonstrate that applying compression through channel attention significantly reduces the total number of model parameters and file size while minimizing loss in detection accuracy. Notably, after compression, performance increases for certain model variants in key AF databases (ADB and C2017DB). Moreover, analyzing the learned channel attention distributions after training enhances the explainability of the AF detection models by highlighting the salient temporal ECG/PPG features most important for its diagnosis. Overall, this research establishes that integrating attention mechanisms is an effective strategy for compressing large DL models, making them deployable on low-power wearable devices. We show that this approach yields compressed, accurate, and explainable AF detectors ideal for wearables. Incorporating channel attention enables simpler yet more accurate algorithms that have the potential to provide clinicians with valuable insights into the salient temporal biomarkers of AF. Our findings highlight that the use of attention is an important direction for the future development of efficient, high-performing, and interpretable AF screening tools for wearable technology.

## 1. Introduction

Early diagnosis of atrial fibrillation (AF), the most common arrhythmia [1], is crucial for preventing stroke and other associated complications [2,3]. While traditional electrocardiogram (ECG)-based diagnosis approaches can be time-consuming, error-prone, and expensive, artificial intelligence (AI) integration in healthcare shows promise in addressing these challenges [4,5]. Machine learning (ML) algorithms can analyze ECG data with potentially high accuracy, automating AF diagnosis and allowing healthcare professionals to focus on other tasks. This improved health data diagnostics also supports cardiologists’ efforts to develop personalized treatment strategies for AF patients. However, realizing AI’s full potential in AF management requires addressing challenges such as data quality, model robustness, computationally efficient implementation, and explainability [5,6]. Despite ML technology still being in its early stages within healthcare, its application for automated AF diagnosis offers a promising avenue for improving both patient care and management efficiency.

Wearable devices annually generate a vast amount of cardiovascular data, including ECG and photoplethysmography (PPG) recordings, which could potentially support first-line medical professionals. However, interpreting ECG data in clinical settings typically requires significant input from cardiology specialists, making the process time-consuming and labor-intensive [7,8]. This challenge is compounded by the fact that paroxysmal AF can be asymptomatic and often goes undetected in short ECG recordings. Consequently, there is a pressing need for tools that can automate this analysis process. Promising developments in this area include ECG-AI, which has demonstrated reliability in clinical conditions [9], and deep learning (DL) algorithms that have shown cardiologist-level potential in arrhythmia detection [10]. These technologies could be applied to wearable devices, potentially revolutionizing the way cardiovascular data are analyzed and interpreted.

As DL models typically have many parameters, a key challenge in automating ECG/PPG analysis for wearable devices is computational efficiency, both in terms of processing power and memory requirements. Another challenge is the explainability of model outputs. This study explores how incorporating an attention mechanism and activation function into a shallow DL model can improve its efficiency and explainability in AF detection. The motivation for introducing model attention stems from neuroscience, where attention mechanisms in the brain concentrate neural processing on the most salient sensory information to achieve an objective and simplify a processing task [11]. Inspired by these aspects, this work implements a channel attention mechanism with a shallow DL model as a relatively simple and computationally low-cost solution to enhance model performance, along with the activation function. The study demonstrates how attention mechanisms may also improve explainability by focusing on salient points in the input RR intervals, potentially supporting future research in AF by simplifying efficient detection algorithms.

This work is organized as follows: A brief review of ML applied to AF detection is given in Section 2. Section 3, Section 4, Section 5, Section 6, Section 7 and Section 8 provide details on the datasets used in this work, the DL channel attention implementation, and the DL model architecture and training.

## 2. Machine Learning for Atrial Fibrillation Detection

Traditional ML approaches for AF detection from wearable devices often involve manual feature extraction, which presents potential limitations. Early methods based on simple peak detection in the time domain focused on heart rate analysis after noise and motion artefact removal [12]. However, this approach relies heavily on the accurate pre-processing of the PPG and accelerometer signals from wrist-worn devices, including time–frequency domain analysis and feature extraction. Additionally, differentiating AF from other irregular rhythms like premature contractions proved challenging due to similar heart rate variations, leading to false positives. More recent studies employ multiple-domain feature extraction, incorporating PPG signals, inter-pulse intervals, and accelerometer data to distinguish AF, atrial flutter, and normal rhythms [13]. Classification then utilizes algorithms such as generalized logistic regression and random forests. While random forests can improve performance through an ensemble of trees, the simpler decision tree approach has also been used with heart rate variability features for AF detection [14]. Similarly, support vector machines (SVMs) have been explored in conjunction with various heart rate features [15].

While traditional ML methods are computationally efficient for wearable devices, they share a crucial drawback: dependence on expert knowledge for selecting optimal features. This reliance on prior expertise limits adaptability and interpretability, and the feature extraction process itself can be computationally demanding. Therefore, alternative approaches that can potentially overcome traditional methods’ limitations are welcome.

DL models, which can automatically learn meaningful features from input data, are gaining traction in AF detection. One DL classifier application involves combining deep convolutional neural networks with remote photoplethysmography (rPPG) extracted from facial video recordings [16]. This approach utilizes data similar to smartphone camera recordings for convenient home use. Another study employed long short-term memory (LSTM) networks, a type of recurrent neural network (RNN) well suited for handling long-term dependencies, to directly analyze raw ECG data [17]. Further advances have combined convolutional neural networks (CNN) and LSTMs to classify AF based on 30 RR interval sections [18]. Residual connection DL models, such as ResNet models, have been used to achieve state-of-the-art results in classifying AF, atrial flutter, and normal rhythm using pre-processed RR intervals [19].

DL models have also demonstrated remarkable performance in demanding medical diagnostic tasks, such as detecting AF in the early stage from sinus rhythm (SR) recordings (raw ECG) [20]. However, the computational complexity of deep learning models can hinder their deployment on resource-constrained wearable devices. One potential solution to address this challenge is the use of interval-based inputs. By leveraging interval-based data, the complexity of the DL models can be reduced, making them more suitable for implementation on wearable edge AI platforms. This approach can help bridge the gap between the superior performance of deep learning and the limited computational resources available on wearable devices.

Despite the higher computational requirements of DL models compared to ML, their ability to achieve superior results directly from raw data makes them a highly attractive option for advancing future AF detection capabilities. By optimizing DL models for wearable platforms, researchers can unlock the full potential of these powerful techniques and drive further improvements in the early detection and management of atrial fibrillation.

## 3. ECG/PPG Databases

Databases were gathered from Physionet [21], which is a collection of multiple databases concerning cardiology, and from the MIMIC-III database [21,22]. The following databases were used for this study: MIT-BIH Atrial fibrillation (AFDB) [23], MIT-BIH arrhythmias (ADB) [24], CinC challenge 2017 (C2017DB) [25], and PPG data of MIMIC PERform AF Dataset (PPGMMAF) [26,27].

**Figure 1:**
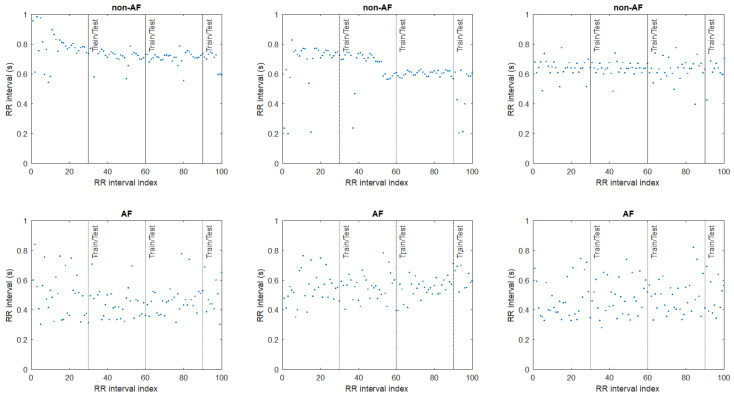
Sequential RR intervals over time (blue dots) extracted from an ECG recording for different patients from AFDB during non-atrial fibrillation (non-AF, **top**) and atrial fibrillation (AF, **bottom**) episodes. The RR intervals have more irregular patterns during AF episodes. A total of 30 RR interval sequences within an episode (vertical lines) were used as input in the DL model (train/test blocks).

AFDB contains 25 patients’ data, mostly from patients with paroxysmal AF. From those, 23 patients have ECG recordings containing two ECG signals and 10-h-long recordings with a sampling frequency of 250 Hz. The recordings were annotated by professional cardiologists: AF, atrial flutter, AV junctional rhythm, and other rhythm episodes in time. The data also include annotations for R peaks, allowing us to calculate RR intervals used in this work (Figure 1). ADB contains data from 47 patients. These include 48 half-hour ECG recordings with a sampling frequency of 360 Hz from patients with various arrhythmias. These data include annotations for arrhythmia episodes and R peak indices [24]. Only patient recordings with sinus rhythm and AF episodes were used in this work. The C2017DB database includes ECG samples of four categories: SR, AF, other arrhythmias, and noisy recordings [25]. It contains a total number of 8528 recordings with a sampling frequency of 300 Hz from which the SR and AF recordings were used. PPGMMAF contains PPG data from 35 critically ill adults during routine clinical care, including data from 19 AF and 16 SR patients, manually annotated by cardiologists. The recordings are 20 min long and were recorded using a sampling frequency of 125 Hz.

## 4. Data Pre-Processing

We chose the RR interval (the interval between consecutive R peaks) as input for training our DL models as it is more efficient to deploy on low-computational devices than raw ECG [28]. Additionally, we tested our model using the PPG signal’s beat-to-beat (BTB) intervals. The RR interval is closely related to the BTB intervals (with some variance) typically used in wearable heart monitoring devices [29]. The beat peaks were processed using an incremental-merge segmentation beat detector from PPG data [30]. The R peaks of C2017DB were extracted using code included in database files.

The recordings from databases were pre-processed and divided into non-AF and AF rhythm labeled episodes for both model training and testing. AFDB recordings were divided into other rhythms and AF episodes (other episodes were excluded). ADB recordings were divided into SR and AF episodes. C2017DB and PPGMMAF data were divided into SR and AF recordings. For each episode 30 sequential RR and BTB intervals were extracted from recordings and used as inputs for the model (Figure 1). If the episode did not include at least 30 intervals, it was excluded.

AFDB data after pre-processing was divided as 76% for training and 24% for testing, while the other databases were used as additional test sets to evaluate model accuracy and generalization across patients before and after compression. After the datasets for training and testing were prepared, the number of recordings of 30 RR and BTB intervals between non-AF and AF label categories was balanced (50/50) by removing the excess from the larger category to mitigate class bias in every dataset. The final data extracted from each dataset are presented in Table 1.

Figure 1 shows typical sequential RR interval differences between non-AF and AF recordings over time. The recordings are divided into 30 RR interval sections for the training and testing sets. The variation in RR intervals within each sequence is significant between the two categories as generally irregularity is seen to increase in the case of AF. However, this irregularity can sometimes occur in non-AF-labeled sequences as well (for example, Figure 1, top right). Therefore, a simple time-invariant feature, such as the RR interval standard deviation, is insufficient for accurate AF classification due to the difficulty in establishing a universal threshold across large populations. As such, a model that can focus on time-varying features as a biomarker for AF is the focus of this study.

## 5. Deep Learning Model Architecture

**Figure 2:**
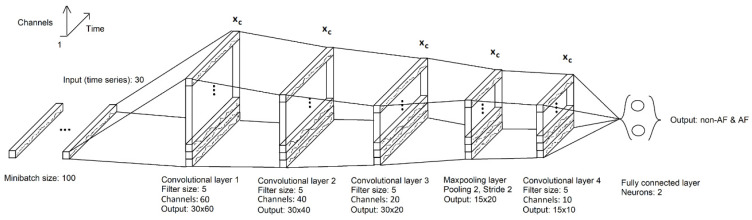
One-dimensional convolutional neural network schematic setup for RR interval classification. The input is a 1D vector of 30 RR interval time series, and convolutional layers use filters (with learnable weights) to form feature maps whose size is successively reduced via pooling. The model output is produced via a fully connected (FC) layer to separate the training data. The convolutional layers are presented with filter size and output size (number nodes and feature maps), and the pooling layer with pooling size.

CNNs are widely used in computer vision tasks, image classification, object detection, and segmentation [31]. One-dimensional CNN variants have also shown excellent performance in time series classification [32] and typically consist of an input layer that takes a fixed-length sequence of the time series (in this case a series of RR intervals), followed by one or more convolutional layers that apply filters to extract temporal features from the input sequence (Figure 2). These learnable filters are adapted to shared weights trained through backpropagation and gradient descent (see Section 7), allowing automatically discover relevant features in the data. This is followed by a pooling layer, which down-samples the feature maps to distil the most salient elements. The flattened output of the pooling layer is then fed into one or more dense (fully connected) layers that interpret the extracted features and produce the final classification output.

The 1D convolution operation between input *x* and weight *w* is calculated as follows:

(1) $$\left(x*w\right)\left[n\right]=\sum_{m=0}^{M-1}x\left[n-m\right]w[M-1-m],$$

where ∗ donates neural convolution and *M* is convolutional operator length [33]. Each neuron within a CNN layer processes information via a non-linear activation function applied to the neuron input, connection weight and a bias:

(2) a=φ(x∗w+b),

where a is the neuron output, ∗ presents neural convolution, x is input and w is the corresponding connection weight, φ is the activation function applied across all neurons, and b is bias. The non-linear activation function is fundamental for mapping input features into an easily separable feature representation of the training data.

## 6. CNN Channel Attention

**Figure 3:**
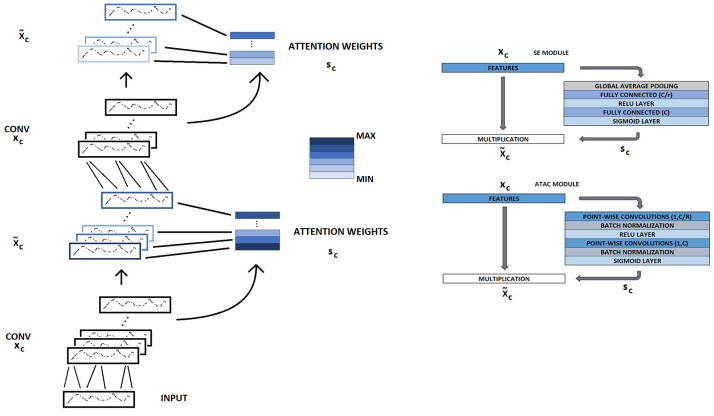
Schematic presentation of the effect of the channel attention mechanism in a compressed CNN model, shown left. The model performance is modified by modulating convolutional layer (CONV) feature maps (channels, $x_{c}$) using attention weights ($s_{c}$). Two distinct attention modules, specifically squeeze–excitation (SE) module [36] and attention-as-activation (ATAC) [37], are employed to generate attention weights, shown right. In the SE module, fully connected layers are configured with neuron counts defined by the ratio of channels to reduction ratio (C/r, r = 3) and the total number of channels (C, varies by layers, see Figure 2). Similarly, in the ATAC module, point convolutional layers with a filter size of 1 are defined by the number of channels, which is a product of the channels per reduction ratio (C/r) and the total number of channels (C). The input to the model is a vector of 30 RR intervals, shown bottom left.

Here, we use channel attention applied to the CNN model to modulate the convolutional layer filter outputs (Figure 3). Channel attention in CNNs is a mechanism that assigns weights to different channels to emphasize or suppress certain features, enhancing the model’s ability to focus on the most relevant features. The combination of channel attention with an activation function makes the mechanism more efficient at focusing on local information which is used in this work to enhance model performance while compressing.

Attention in neural networks is inspired by the neuroscience observation that humans selectively focus on certain aspects of information while ignoring others, akin to the visual attention system where the eye scans an image, focusing on specific regions with higher resolution to form a more detailed representation of the target [34]. Similarly, the attention mechanism in neural networks allows the model to focus processing on the most salient parts of the input. This mechanism has gained significant interest in recent years as it has been shown to improve the performance of various deep learning models [11,35,36,37]. The attention mechanism can be categorized based on the type of information it focuses on, such as what, where, when, or which to pay attention to, with channel attention identified as the most computationally efficient method [36,38].

The channel attention is implemented in our 1D CNN using a squeeze–excitation (SE) sub-network [36], based on scalar multiplications with feature maps generated by the CNN layers:

(3) $${\tilde{X}}_{C}=F_{s}\left(x_{c,}s_{c}\right)=s_{c}x_{c},$$

where ${\tilde{X}}_{C}$ and $F_{s}$ indicate channel-wise multiplication between a scalar ($s_{c}$) and CNN feature map $x_{c}$ [36]. The scalar is formed using global pooling, two fully connected (FC) layers, and activation functions ReLu and sigmoid using the following equation:

(4) $$s_{c}=\sigma \left(W_{2}\delta \left(W_{1}z\right)\right),$$

where σ represents the sigmoid activation function, δ represents ReLu activation, $W_{1}$ and $W_{2}$ are fully connected layers, and z is input via global average pooling. The fully connected layer size is determined by feature layer (input) size and reduction rate *r*.

For comparison, we also used the attention as activation (ATAC) module to study the effect of the local channel-wise attention mechanism in addition to scalar-based attention. The ATAC module combines activation function and attention mechanisms that have been shown to be more efficient [37]. It uses two point-wise convolution layers with batch normalization (BN), ReLu, and gating function:

(5) $$L(x_{c})=\alpha (W_{PWConv2}\delta (W_{PWConv1}x_{c})),$$

where $W_{PWConv1}$ and $W_{PWConv2}$ are point-wise convolutions with batch normalization, δ is the ReLu activation function, and α is the gating function [37]. It is also used to emphasize specific channels via multiplication with feature maps:

(6) $$X^{\prime }=L\left(x_{c}\right)⊙x_{c},$$

where $L\left(x_{c}\right)$ represents the output of the gating module, ⊙ is an element-wise multiplication operator, and $x_{c}$ is a feature map. The multiplication between the gating function and feature maps is the module’s output, but also the activation function output of the layer. However, this work uses only a sigmoid gating function similar to SE module to provide a local channel attention mechanism without replacing the activation function.

## 7. Model Training

We used cross-entropy as the loss function, which is the total entropy between prediction distributions of the output (between 0 and 1) defined as follows:

(7) $$L\left(o,y\right)=-\sum_{j}y^{\left(j\right)}\log\sigma {\left(o\right)}^{\left(j\right)},$$

where y represents the true label (non-AF or AF), and o is the output of the network’s final layer. We used a stochastic gradient decent (SGD) (minibatch), which is especially computationally efficient for large datasets as it does not require computing gradients over the entire dataset but instead a minibatch in each iteration:

(8) $$\theta =\theta -\eta \cdot \nabla_{\theta }J\left(\theta ;x^{\left(i:i+n\right)};y^{\left(i:i+n\right)}\right),$$

where θ presents the parameters, η is the learning rate, $x^{\left(i:i+n\right)}$ is the training examples, and $y^{\left(i:i+n\right)}$ is the corresponding label. Furthermore, the minibatch strategy gradient decent enables more stability during the training of SGD [39]. The best result for our baseline CNN (see Section 8) was obtained by setting the initial learning rate to 0.0001, minibatch size to 100, and epochs to 25. These hyperparameters tuned for baseline were also used with the compressed models.

**Figure 4:**
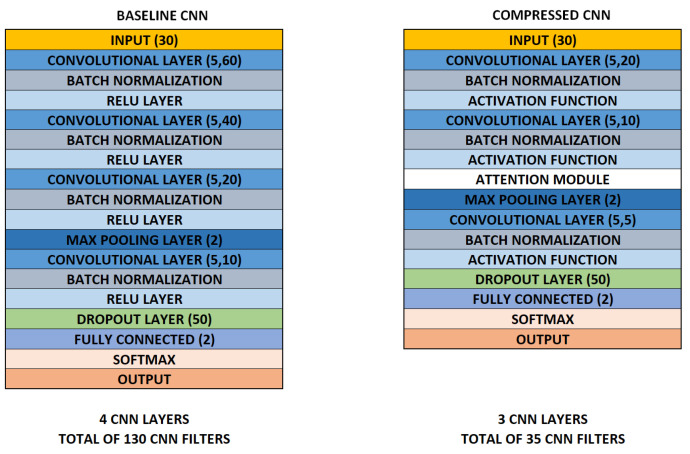
The baseline (**left**) and compressed CNN with attention modules added (**right**). The baseline CNN has four convolutional layers, whereas the compressed version has three smaller layers with an attention mechanism—either an SE or ATAC module.

After training the baseline model, the CNN model was compressed and customized by adding SE and ATAC modules. The compressed models were then trained separately. The models were trained using attention mechanisms in every CNN and only in one layer. The best results from these setups are presented in this work, which were achieved by applying an attention mechanism only in one layer and a reduction rate *r* = 3 (Figure 3). The baseline CNN model (Figure 4, left) contains four 1D convolutional layers with feature maps of 60, 40, 20, and 10 (filter size of 5). Each layer includes batch normalization and ReLu activation functions. A max-pooling layer (2 with stride 2) was applied between the third and fourth layers. Furthermore, the fourth layer includes dropout (50%). The dropout layer randomly drops the output of neurons during training to avoid overfitting. The last layers are fully connected layer (2) and Softmax.

The compressed models (Figure 4, right) contained three 1D convolutional layers with feature maps of 20, 10, and 5 with a filter size 5 (batch normalization in each layer). Three compressed models were CNN models combined with SE attention module using ReLU, Swish, sine activation functions (CNNrl-S, CNNsw-S, and CNNsn-S, respectively). Furthermore, another three models were combined with the ATAC attention module using the same activation functions (CNNrl-A, CNNsw-A, and CNNsn-A, respectively). The max pooling layer was applied between the second and third CNN layers and the dropout in the third layer. Matlab was used as API for training using a personal laptop.

## 8. Results

The baseline CNN was trained using multiple trials with varying compression model choices. The trained models were evaluated using accuracy (Ac) and F1 measure (Table 2). Furthermore, sensitivity (Sen) and specificity (Spec) were used with the AFDB test set.

**Figure 5:**
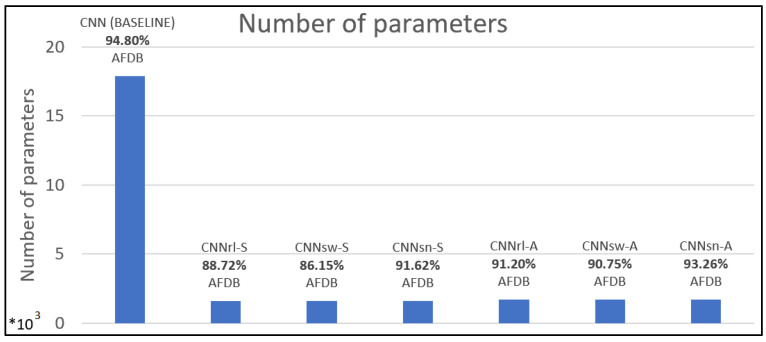
The number of parameters of models with AFDB test set accuracy.

**Figure 6:**
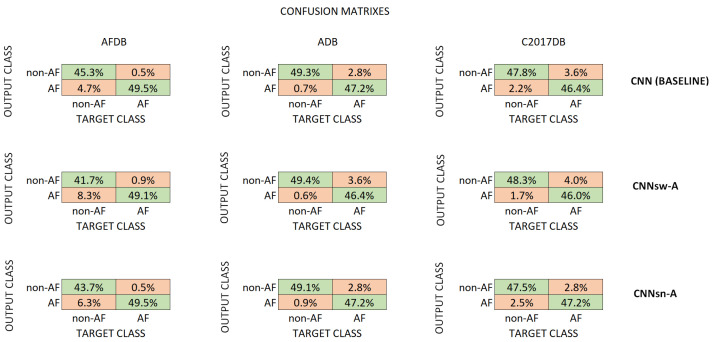
Confusion matrixes of the trained models (green correct, red incorrect class predictions). On the left are AFDB, in the middle ADB, and on the right C2017DB matrixes. The top row presents baseline CNN model performance, the middle model with ATAC with Swish, and the bottom model with ATAC with sine.

Table 3 shows the number of parameters and file size to evaluate the computational requirements for the models. Figure 5 shows the number of parameters of the model, along with the test accuracy for AFDB, and Figure 6 presents the confusion matrixes of a selection of trained models (across test sets). The compressed models’ parameters decreased by ~91% compared to the baseline model (17,900). Furthermore, file size decreased by ~56% compared to the baseline model (104 kB). The number of parameters is important in low-computational devices like edge technology [28].

When the SE module was applied to the model, the highest accuracy for AFDB was 91.62% (CNNsn-S), 3.18 percentage points less than the baseline accuracy of 94.80%. The accuracy of 93.27%, 1.53 percentage points less than the baseline, was highest when the ATAC module was applied to the model (CNNsn-A). The sensitivity of 98.36% (CNNsn-S), 0.71 percentage points less than the baseline, was highest among models with the SE module. The highest specificity of the same models was 84.87% (CNNsn-S), 5.65% percentage points less than the baseline. The sensitivity of 99.07%, the same as the baseline, was highest among the models with the ATAC module (CNNsn-A). The same model’s highest specificity was 87.47% (CNNsn-A), 3.05 percentage points less than the baseline (Table 2). The highest accuracy for ADB (97.88%) was achieved using CNNrl-A. The baseline model’s accuracy was 96.58% less than all other compressed models’ accuracy except CNNsw-A. For the C2017DB test set, the highest accuracy was 95.62% using CNNsn-A. The baseline accuracy was 94.14% less than three compressed models (CNNsn-A, CNNsw-A, and CNNsn-A).

Table 4 presents test results using PPG data. The highest accuracy using PPG data for models with SE module was 89.34%, achieved using a model CNNsn-S and with models with the ATAC module 90.05% using CNNsn-A. The baseline model accuracy was 90.77%. The results show that there is no significant reduction in the accuracy of the compressed models compared to the baseline, which is the main focus of our work.

**Figure 7:**
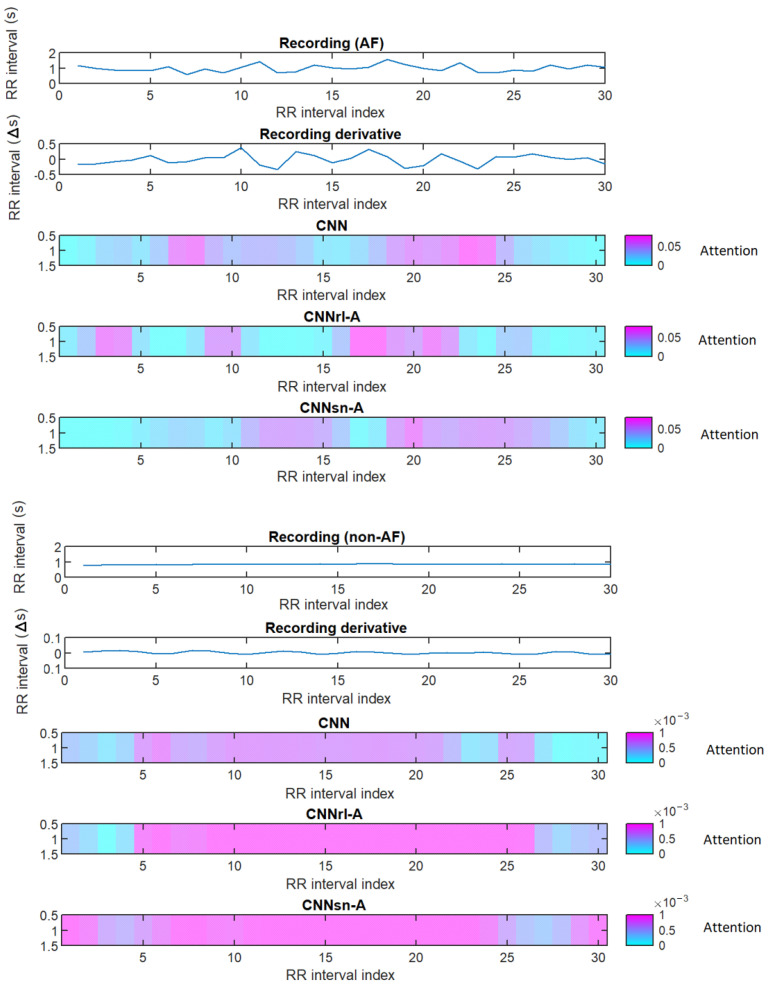
Gradient-weighted class activation mapping map of the change in the classification score as a measure for network attention focus, for example, AF (**top**) and non-AF (**bottom**) recordings. Below each recording is the attention focus to the RR intervals over time for various models against the RR interval derivative.

To understand how attention is being used by the models, Figure 7 shows the gradient-weighted class activation mapping map of the classification score change based on the classification score’s gradients for the final convolutional layer for two example recordings (non-AF and AF). It shows at which point of a recording the models attend the most. Using an attention mechanism and choice of activation function, both change the attention focus compared to the baseline model. In the AF example (Figure 7, bottom) we see that CNNrl-A attention modulation correlates with RR interval changes seen in the derivative plot. Furthermore, the choice of activation function had a significant effect on changing that focus.

To check the importance of attention in compressed model performance, an ablation study was carried out by removing the attention modules. The compressed models with ReLu, Swish, and sine activation functions were used in this study (CNNrl, CNNsw, and CNNsn). The results from the ablation study are presented in Table 5 and Table 6. The accuracy of the AFDB test set showed a decrease with all activation functions compared to the models with attention modules (Table 2). The same trend was also observed using PPG data (Table 4 and Table 6). The ADB and C2017DB accuracy showed more variations in the results. Thus, the model performance was seen to be reduced when attention modules were removed.

We also checked the compressed and uncompressed model classification performance using 5-fold cross-validation on the training set, presented in Table 7. The highest accuracy among the compressed models was achieved using CNNsn-A (97.26%). The baseline accuracy was 98.04%.

## 9. Discussion

AF is a prevalent cardiac arrhythmia that poses a significant public health challenge worldwide, underscoring the critical importance of developing accurate and reliable methods for its detection to improve patient outcomes and reduce the burden on healthcare systems globally. The accuracy of AF detection plays a crucial role in its management, and DL offers the potential for automated end-to-end learning of time-series features in both non-AF and AF recordings. However, a major challenge arises when deploying DL models on wearable devices such as smart devices, which typically have limited computational power. DL models normally require many parameters and substantial computational resources, making it difficult to execute efficiently on edge AI hardware. To address this challenge, this work studied a compressed convolutional neural network (CNN) model with customized channel attention and activation function. Overall, our results show the potential for compressed DL models for AF detection using ECG/PPG data, which is important for low-computational devices such as wearables.

The combined CNN attentional network achieves comparable performance to state-of-the-art results on the AFDB dataset using RR intervals as input (Table 8). Previous DL works for AF detection, in comparison, have used larger models with significantly more parameters (e.g., [40] 343,301 parameters, [18] 159,841 parameters, and [41] estimated more than 17,900 parameters more than what our models required—Table 3). For example, long-short-term memory (LSTM) was developed to fix the vanishing gradient problem with recurrent neural networks [42], but these are computationally costly, and efficiency improvements have since been made [43,44,45].

ML approaches, while generally simpler in complexity, rely on hand-crafted features that may or may not generalize to different contexts, and these studies report lower performance with AFDB overall. In this work, we found that the combination of the channel attention mechanism with an activation function significantly enhanced the CNN performance compared to the baseline model while retaining automated feature discovery and reducing the number of parameters overall.

Attention is a powerful technique in DL that improves model performance in several ways:

- Attention improves a model’s ability to focus on the most relevant parts of the input data. Our results show that incorporating attention allows models to focus on irregularity in RR intervals, which are important for accurate AF detection.
- Channel attention enhances interdependence between channels, discriminating features and suppressing noise, as shown in previous work on arrhythmia detection [48]. Our results show improved model accuracy in AF detection across test sets.
- Using channel attention in this context improved efficiency of the training by optimizing layer-wise feature representation to encode higher-level semantics more efficiently [36,37].
- By focusing network processing capacity attention simplifies the processing overall and may be used as an approach to discover accurate yet simple algorithmic solutions to AF detection and guide clinicians in ECG interpretation.

While attention has been incorporated into DL models applied to raw ECG data previously [48], this work demonstrates its effectiveness with compressed models when using RR interval data that is particularly well suited to low-computational devices such as wearables.

We found that the choice of activation function plays an important role in model performance. The form of non-linearity introduced by the activation function enables the network to discover complex relationships in the data and channel information. ReLu (and its variants) is typically used because of its simplicity and relatively low computational cost. However, there exist many other choices [49]. For example, Swish was used in this work to avoid the ReLu vanishing gradient problem due to zero values for negative inputs [50]—instead containing a slope near zero to address this issue. TanSoft activation functions have similar properties [51] and show improved performance compared to ReLu but depend on learnable parameters that can be a drawback since while additional learnable parameters can make the function more adaptive to data, these need to be optimally set to avoid overfitting. Another choice, TanSoft contains multiple core elements similar to Mish, which might limit its use in compressed DL models [49]. PAU is another activation function with learnable parameters with the same limitations. For low-computational devices such as wearables, exponents in the activation functions should also be avoided as they can further increase the computational cost. Periodic activation functions with compressed DL models for AF detection demonstrated good performance in our study. Compared to commonly used ReLu, the periodic activation functions are non-local, providing opportunities for representation of the data and learning channel interrelationships [52]. In our work, we found that a periodic activation function (when combined with the attention mechanism) improved the model’s accuracy to previously seen and unseen datasets.

Explainability methods have become an important aspect of DL models, assisting users and developers in understanding and trusting the models’ decision-making processes, which is important with wearable device-integrated models. The improved explainability potentially helps to recognize other arrhythmia episodes and new elements of the RR interval data relevant to other conditions, which cannot be easily separated from ECG/PPG recordings. For example, atrial flutter, which is similar to AF but less common, is important to discriminate against because optimal treatment options differ [13]. Our results show that depending upon the choice of attention and activation function, DL networks focus on RR interval changes in detecting AF, providing a form of explainability for the classification outcomes.

Finally, our study showed potential for PPG data processing, even when trained on ECG-derived RR interval data. Since we trained on ECG data from a range of devices and tested against PPG data, which should be equivalent to data expected from a wearable device, e.g., smartwatches, our results demonstrate the degree to which the DL models may be applied to a realistic wearable device. In the future, further accuracy improvements could be obtained in wearable devices by training exclusively on PPG data. Our results also indicate that it can improve model performance on PPG data using ECG data for training, which would be an interesting aspect to study more in the future because the PPG recordings can contain more noise. Further work should focus on training and testing with PPG data that are only common in wearable devices.

Future work should further explore the role of attention mechanisms for efficient and explainable AF detection [34], and the use of specialized hardware accelerators or low-power neural processing units (NPUs) to further reduce the computational requirements of the compressed models (e.g., TinyML), which will continue to be important in wearable devices. Furthermore, exploring techniques for on-device model updating and personalization could enhance the models’ adaptability to individual variations and improve overall performance. Finally, one promising area of investigation could be the integration of multimodal data sources, such as combining ECG/PPG data with other sensor data available on wearable devices (e.g., accelerometer and heart rate variability). This multimodal approach may provide additional context and improve the robustness of AF detection, particularly in noisy or challenging environments.

## 10. Conclusions

This study presents a novel model compression method for DL models, including existing lightweight models. The proposed technique not only compresses the model but also enhances its performance, making it a viable alternative to other compression methods, such as distillation, pruning, and quantization, which often compromise model performance.

Low-computational devices, such as wearable devices, can play a significant role in the future concerning AF management. For example, early detection would provide a better success rate in treating arrhythmia. This study proposes a novel approach by integrating a customized attention mechanism into a DL neural network. Channel attention allows the model to focus only on the most salient time-series features, thereby reducing the computational requirements. The results of this study demonstrate that by applying compression techniques such as channel attention, the number of model parameters, training time, and file size can be significantly reduced. Importantly, the loss in detection accuracy is minimized and increases for certain model variants. Furthermore, the study highlights the potential of analyzing channel attention after training to enhance the explainability of DL models. This has implications for the development of simpler and more accurate AF detection algorithms, as well as providing valuable insights to clinicians regarding the salient temporal features relevant for AF detection. Overall, this research demonstrates that integrating attention mechanisms can be an effective strategy for compressing large-parameter DL models, making them suitable for deployment on target devices with low computational power. The implications of this work are relevant for developing computational solutions for healthcare applications, such as AF detection, especially in resource-constrained settings and on low-computational devices. In summary, the integration of low-computational devices, particularly wearable devices, with artificial intelligence holds immense promise for improving AF management through automated detection, a crucial step towards enhancing treatment quality and preventing arrhythmia progression.

## 11. Limitations

The compressed models were not tested using live data. The live data of ECG/PPG can contain more noise, and therefore, can cause incorrect outcomes. Testing these models with live data is crucial to ensure their robustness and accuracy in practical scenarios.

## Figures and Tables

**Table 1:** Data amount extracted from each database for final dataset used for training and testing of our DL model (RR and BTB intervals). Non-atrial fibrillation (non-AF) and atrial fibrillation (AF) labels (50/50).

Database	Intervals (RR and BTB) Non-AF and AF (50/50 per Class)
AFDB (train)	760,620
AFDB (test)	186,780
ADB (test)	25,440
C2017DB (test)	50,700
PPGMMAF (test)	46,140

**Table 2:** Model performance on AF/non-AF classification of baseline and compressed CNN models. ‘*’ indicates models accuracy outperformed baseline accuracy.

Model	Ac % (AFDB)	Sen % (AFDB)	Spec % (AFDB)	F1 % (AFDB)	Ac % (ADB)	F1 % (ADB)	Ac % (C2017DB)	F1 % (C2017DB)
CNN (baseline)	94.80	99.07	90.52	95.01	96.58	96.49	94.14	94.06
CNNrl-S	88.72	98.07	79.38	89.69	97.41 *	97.39 *	93.02	92.95
CNNsw-S	86.15	97.56	74.75	87.57	96.70 *	96.66 *	92.43	92.34
CNNsn-S	91.62	98.36	84.87	92.15	96.70 *	96.59 *	95.62 *	95.58 *
CNNrl-A	91.20	98.97	83.42	91.83	97.88 *	97.86 *	93.91	93.87
CNNsw-A	90.75	98.10	83.39	91.38	95.99	95.83	94.32 *	94.19 *
CNNsn-A	93.27	99.07	87.47	93.64	97.05 *	93.64	94.67 *	94.66 *

**Table 3:** Number of parameters, training time, and file size of the model.

Model	Number of Parameters	File Size
CNN (baseline)	17,900	104 kB
CNNrl-S	1600	47 kB
CNNsw-S	1600	46 kB
CNNsn-S	1600	47 kB
CNNrl-A	1700	46 kB
CNNsw-A	1700	46 kB
CNNsn-A	1700	48 kB

**Table 4:** Test results using PPG data.

Model	Accuracy %	F1 %
CNN (Baseline)	90.77	90.71
CNNrl-S	85.89	85.65
CNNsw-S	86.54	86.35
CNNsn-S	89.34	89.23
CNNrl-A	88.35	88.22
CNNsw-A	89.47	89.45
CNNsn-A	90.05	89.96

**Table 5:** Test results of attention ablation study.

Model	Ac % (AFDB)	Sen % (AFDB)	Spec % (AFDB)	F1 % (AFDB)	Ac % (ADB)	F1 % (ADB)	Ac % (C2017DB)	F1 % (C2017DB)
CNNrl	84.90	96.62	73.17	86.48	97.76	97.75	93.25	93.17
CNNsw	83.85	95.34	72.37	85.52	95.87	95.83	93.55	93.43
CNNsn	89.95	98.20	81.68	90.71	96.93	96.85	95.50	95.49

**Table 6:** Test results of PPG data after attention ablation study.

Model	Accuracy %
CNNrl	86.67
CNNsw	82.24
CNNsn	85.69

**Table 7:** Cross-validation results for 5-fold cross-validation.

Model	5-Fold Accuracy %
CNN (baseline)	98.04
CNNrl-S	96.21
CNNsw-S	95.62
CNNsn-S	97.16
CNNrl-A	96.56
CNNsw-A	95.51
CNNsn-A	97.26

**Table 8:** Comparison between ML/DL research works for AF detection using AFDB and RR interval inputs.

Method	Input	Accuracy
CNN-BiLSTM, [18]	RR intervals	97.80%
CNN-BiLSTM, [41]	RR intervals and heartbeat sequences	96.59%
BiLSTM, [40]	RR intervals	98.51%
SVCm, [46]	RR intervals	95%
Entropy measure, [47]	RR intervals	93.51%
Proposed method, 2024	RR intervals	97.26%

## Data Availability

The data presented in this study are available from the corresponding author upon request.
